# Identification and Expression Analysis of the *bHLH* Gene Family in *Rhododendron* × *pulchrum* Sweet with Different Flower Colors

**DOI:** 10.3390/plants14111713

**Published:** 2025-06-04

**Authors:** Jiaran Sheng, Jianshang Shen, Yingying Shan, Xia Chen, Xueqin Li, Huasen Wang, Songheng Jin

**Affiliations:** 1 College of Horticulture Science, Zhejiang A&F University, Hangzhou 311300, China; 18888749909@163.com (J.S.); whsych66@163.com (H.W.); 2 Jiyang College, Zhejiang A&F University, Zhuji 311800, China; syy15968566686@163.com (Y.S.); cx2912@zafu.edu.cn (X.C.); lxqin@zafu.edu.cn (X.L.); 3 College of Horticulture, Hangzhou Vocational & Technical College, Hangzhou 310018, China; shenjianshuang18@163.com; 4 College of Horticulture, Qingdao Agricultural University, Qingdao 266109, China

**Keywords:** *Rhododendron* × *pulchrum* Sweet, *bHLH* family, transcription factors, bioinformatics, gene expression, flower development, flower color

## Abstract

Basic helix–loop–helix (*bHLH*) transcription factors play significant roles in plant growth and organ development and diverse biochemical processes. However, the function of *bHLH* transcription factors in woody plants is not fully understood. In this study, the *bHLH* gene family in *Rhododendron* × *pulchrum* Sweet was identified and characterized using whole-genome data. A total of 109 *bHLH* family genes (*RpbHLHs*) were identified in *R*. *pulchrum*, and their expression levels were analyzed in flowers of different colors and developmental stages. The results showed that the *RpbHLH* family is divided into 24 subfamilies. Chromosomal localization and collinearity analyses revealed numerous duplication events during evolution, which is one of the main reasons for the diversification of gene functions. The bHLH domains showed relative conservation of RpbHLH proteins. In the promoter regions of the *RpbHLHs*, various cis-regulatory elements involved in light response, gibberellic acid (GA) response, and abscisic acid (ABA) response were identified. These elements may regulate flower development and pigment synthesis. A Kyoto Encyclopedia of Genes and Genomes (KEGG) functional enrichment analysis of the target *RpbHLHs* revealed that 25 genes are enriched in the flavonoid biosynthetic pathway. Potential *RpbHLH*s related to flower development and pigment synthesis were identified through a transcriptome analysis and validated through quantitative reverse transcription PCR (qRT-PCR). This study will enhance our understanding of *RpbHLH* functions and provide a reference for the study of flower development and coloration in *R*. *pulchrum*.

## 1. Introduction

*Rhododendron* × *pulchrum* Sweet (*R*. *pulchrum*) belongs to the *Rhododendron* genus in the Ericaceae family. As an alpine flower, it has been extensively cultivated in China for a considerable period. Recognized as one of China’s top ten most famous flowers, *R*. *pulchrum* boasts lush foliage and vibrant blooms, making it a popular choice for landscaping [1]. In addition to its ornamental value, *R*. *pulchrum* possesses significant economic and medicinal utility. Its leaves are used for fragrance extraction, while the petals serve as a source of essential oils. The chemical compounds derived from this plant find applications as cardiovascular, anesthetic, and antimalarial medications [1].

The basic helix–loop–helix (*bHLH*) transcription factor is the largest family of transcription factors in plants [2]. The *bHLH* transcription factor has a highly conserved basic helix–loop–helix domain [3], which consists of 50–60 amino acids and is divided into two regions: the N-terminal basic region and the C-terminal α-helix 1-loop-α-helix 2 (HLH) region [2]. The basic region contains 15–20 conserved amino acids, which determine the recognition of the core E-box (5′-CANNTG-3′) and the binding of *bHLH* transcription factors to DNA [3]. The HLH region is composed of two hydrophobic residues linked by a more diverse loop region, allowing the HLH domain to facilitate protein–protein interactions and form homo- or heterodimer complexes, giving rise to two amphipathic α-helices separated by a loop of a variable length [2,4,5]. Previous studies have analyzed the whole-genome sequences of nine terrestrial plants and algae, classifying the *bHLH* gene family into 15–26 subfamilies [2,4]. Many *bHLH* gene families have been identified in *Arabidopsis*, *Prunus mume* (*P*. *mume*), *Passiflora edulis* (*P*.*mume*), *Solanum lycopersicum* (*S*. *lycopersicum*), and *Malus domestica* [6,7,8,9,10]. However, the identification of the *bHLH* (*RpbHLH*) gene family in *R*. *pulchrum* has not been reported.

In anthocyanin biosynthesis, *bHLH* transcription factors act as key regulators. Anthocyanins, one of the major pigments in plants, determine flower color diversity. *bHLH* transcription factors regulate key genes in the anthocyanin biosynthesis pathway, directly influencing flower color formation. Specifically, *bHLH* transcription factors form complexes with other transcription factors such as *MYB* and *WD40* to regulate the expression of genes involved in anthocyanin biosynthesis, including CHS (chalcone synthase), F3H (flavanone 3-hydroxylase), and DFR (dihydroflavonol 4-reductase), thereby promoting anthocyanin accumulation and influencing flower color [11]. For instance, in *Arabidopsis*, *bHLH* transcription factors such as TT8 interact with *MYB-type* transcription factors like *MYB75*, forming a complex that activates the expression of key genes in the anthocyanin biosynthesis pathway, thereby promoting anthocyanin accumulation and influencing flower color formation [12].

In addition to flower color regulation, *bHLH* genes also play crucial roles in flower development. Studies have shown that *bHLH* transcription factors regulate the development of various plant organs, including anther and carpel development. In *Arabidopsis*, the *bHLH* transcription factor *SPATULA*, expressed in the anthers, regulates carpel development [13]. In rice, *UDT1* regulates anther development and is essential for tapetum and microspore maturation [14]. By regulating downstream target genes, *bHLH* transcription factors promote the normal development of flowers, ensuring the correct formation and function of the floral organs.

Although *bHLH* genes have been well studied in many plants and their roles in flower development and color regulation are well established, the functional roles of the *bHLH* gene family in woody plants, particularly in the genus *Rhododendron*, remain underexplored. This study aims to systematically identify the *bHLH* gene family in *R. pulchrum* through a genome-wide analysis and to explore their roles in flower development and color regulation through a gene expression analysis. We combined gene expression data to investigate further how *bHLH* genes influence flower color by regulating the anthocyanin biosynthesis pathway and explored their potential roles in flower development. In addition, this study lays the foundation for future functional validation experiments, including the identification of specific pigments involved in flower colors, and aims to provide valuable theoretical support for understanding the regulatory mechanisms of *bHLH* genes in flower development. Ultimately, this research not only contributes to the identification of *bHLH* genes related to flower colors in *R. pulchrum* but also offers a foundation for further functional studies and applications in horticultural practices and ornamental plant breeding.

## 2. Results

### 2.1. Flower-Color- and Stage-Specific Variations in Flavonoid and Anthocyanin Contents

**Figure 1:**
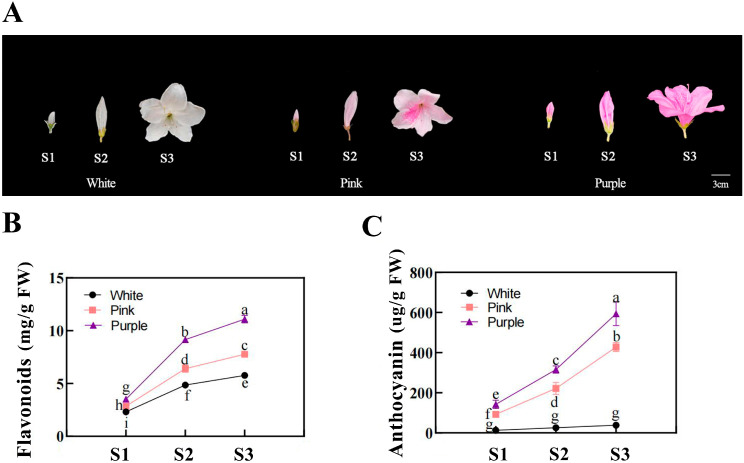
Flower development and pigment analyses of different flower colors in *Rhododendron simsii* at various stages. (**A**) Photographs of *Rhododendron simsii* flowers at three developmental stages—the bud stage (S1), the flowering stage (S2), and the fully open flower stage (S3)—for white, pink, and purple flower colors. Scale bar: 3 cm. (**B**) The flavonoid content (mg/g) in flower petals at different developmental stages (S1, S2, S3) for white, pink, and purple flowers. Different letters indicate significant differences between stages within each flower color (*p* < 0.05). (**C**) Anthocyanin content (µg/g) in flower petals at different developmental stages (S1, S2, S3) for white, pink, and purple flowers. Different letters indicate significant differences between stages within each flower color (*p* < 0.05).

Distinct pigmentation patterns were observed among flower colors during development (Figure 1). From the bud stage (S1) to the fully open flower stage (S3), both the flavonoid and anthocyanin contents increased continuously in purple flowers. Pink flowers also showed an increasing trend in their pigment content, though at significantly lower levels than those in purple flowers. In contrast, white flowers maintained low levels of flavonoids and had almost undetectable anthocyanin levels throughout development.

### 2.2. The Identification of the RpbHLH Family and an Analysis of the Physicochemical Properties of RpbHLH Proteins

To screen and identify the *bHLH* gene family in *R. pulchrum*, Hmmscan was employed to align with the PFAM database. A total of 109 candidate genes encoding basic helix–loop–helix (bHLH) proteins (RpbHLH) were selected from the genomic database of *R. pulchrum* and subsequently renamed *RpbHLH01–RpbHLH109*.

The basic features, including the amino acid count, protein molecular weight, isoelectric point, coding sequence length, grand average of hydropathicity, aliphatic index, and subcellular localization, were analyzed for the RpbHLH proteins identified (Table S1). Among the 109 RpbHLH proteins, the average amino acid count was 397. The molecular weight of the RpbHLH proteins ranged from 1.82 kDa (for RpbHLH105, which had the lowest amino acid count of 163) to 16.03 kDa (for RpbHLH99, which had the highest amino acid count of 1407). The isoelectric points were between 4.48 (RpbHLH80) and 10.37 (RpbHLH95), averaging 6.73. The longest coding sequence was found in *RpbHLH99,* with 4221 nucleotides, while the shortest was in *RpbHLH105,* with 489 nucleotides. The grand average of hydropathicity values were all negative, indicating that all of the RpbHLHs were predominantly hydrophilic proteins. The aliphatic index was between 46.87 (*RpbHLH61*) and 103.9 (*RpbHLH72*). In terms of their subcellular localization, a total of 98 of the RpbHLH proteins were found to be predominantly localized in the nucleus, with 4 proteins detected in the cytoplasm. Additionally, two proteins were associated with the chloroplasts, and one protein each was identified in both the nucleus and the cytoplasm, the Golgi apparatus, the endoplasmic reticulum, the vacuole, and the peroxisome.

### 2.3. The Phylogenetic Analysis of the RpbHLHs

**Figure 2:**
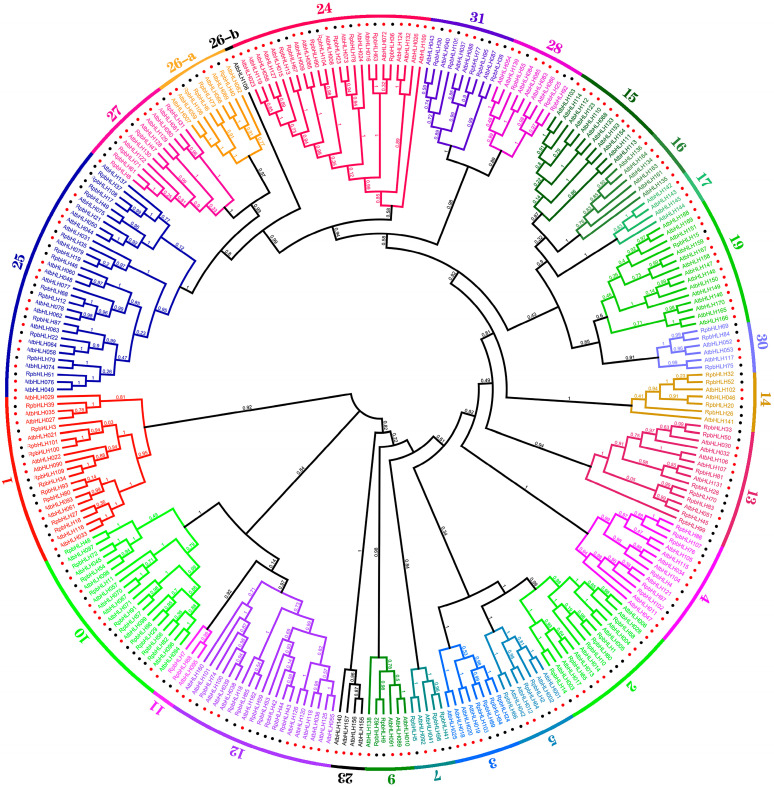
The maximum likelihood phylogenetic tree of bHLH proteins from *R. pulchrum* and *Arabidopsis thaliana*. The tree was constructed using FastTree software. Branches of different colors represent distinct subfamilies. The numbers in the outer circles denote the subgroup names. The red dots represent *Arabidopsis* genes, and the black dots represent *Rhododendron* genes.

To investigate the evolutionary relationships of the *Rhododendron* × *pulchrum* Sweet (*RpbHLH*) gene family further and classify its subfamilies, the protein sequences of the 109 identified *RpbHLHs* were aligned with 166 *Arabidopsis* bHLH proteins (*AtbHLHs*) using Mafft v7.313 and FastTree 2.1.11 to construct a phylogenetic tree. As shown in Figure 2, the 275 *bHLH* genes of *R*. *pulchrum* were grouped into 31 subfamilies according to the taxonomic classification proposed in previous studies [5,15], with the subfamily numbers ranging from 1 to 31. The distribution of the 109 *RpbHLH*s across the subfamilies was uneven. Subfamily 25 contained the highest number of genes (14), while subfamily 19 included only a single gene (*RpbHLH*). Notably, no *RpbHLH*s were found in subfamilies 6, 8, 18, 20, 21, 22, or 29, suggesting the potential loss of these subfamilies during the evolution of *R*. *pulchrum*. Subfamilies 15, 16, 17, and 23 were exclusively composed of *AtbHLH* genes, while the remaining subfamilies contained a mixture of both *RpbHLH* and *AtbHLH* genes, indicating species-specific differences in gene family evolution.

These findings reveal significant evolutionary divergence between *Rhododendron* × *pulchrum* Sweet and *Arabidopsis thaliana*, potentially linked to the unique flower color characteristics of *R*. *pulchrum*. The clustering of *bHLH* genes into specific subfamilies suggests that certain subfamilies may be involved in regulating flower color and developmental stages. The uneven distribution of the *RpbHLH*s across these subfamilies, with some subfamilies containing only *R*. *pulchrum*-specific genes, suggests that these genes may specialize in controlling flower pigmentation and developmental processes. This study underscores the crucial role of the *bHLH* gene family in regulating both flower color and developmental stages in *R*. *pulchrum*. Specific subfamilies may influence the formation of different flower colors, as illustrated in Figure 2. A further analysis of the expression patterns of these genes across various developmental stages and flower color morphs will provide deeper insights into the molecular mechanisms underlying flower color variations in *R*. *pulchrum*.

### 2.4. Analysis of RpbHLH Structures

**Figure 3:**
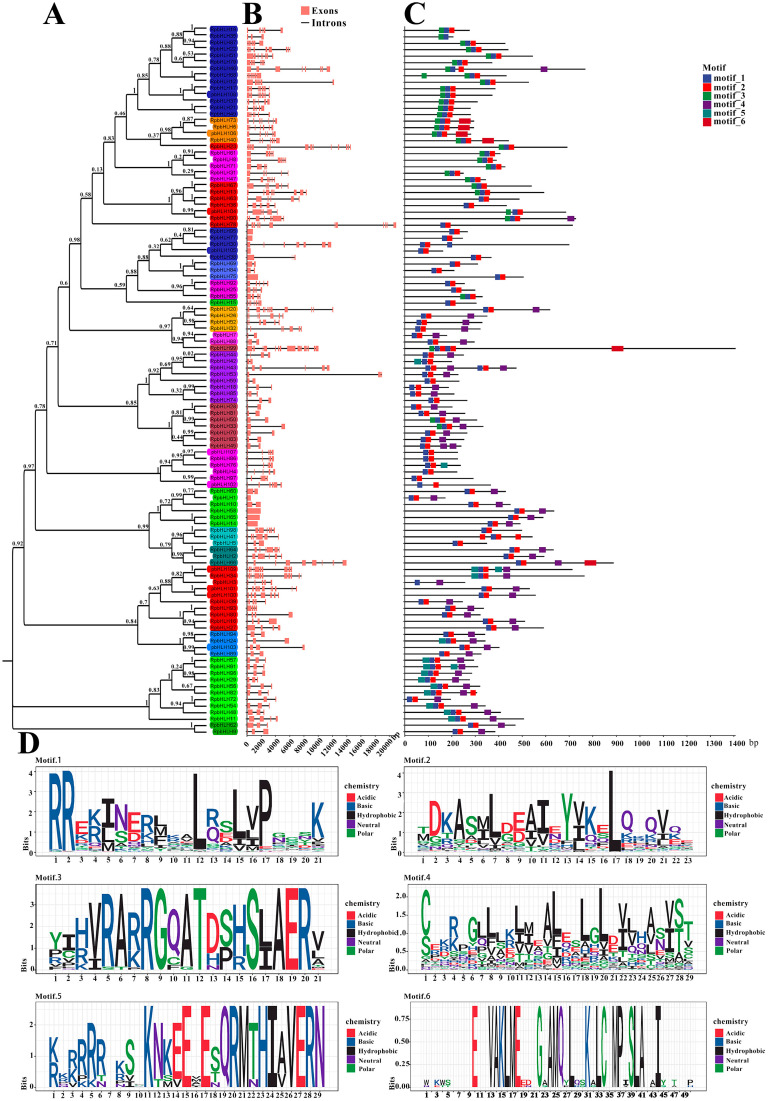
The phylogenetic relationship, conserved motifs, and gene structure of *RpbHLHs*. (**A**) A neighbor-joining phylogenetic tree of the *RpbHLHs*. (**B**) The gene structures of *RpbHLHs*, including exons (pink boxes) and introns (black lines), with the gene lengths displayed at the bottom. (**C**) Identification of conserved motifs in *RpbHLHs* using MEME software; six patterns were identified. (**D**) Display of sequences of conserved Motifs 1–6: motif 1 consists of an α-helix 1 region and half a loop region; motif 2 contains an α-helix 2 region and a loop region; motif 3 represents an acidic region; and motifs 4–6 denote non-conserved regions.

First, a phylogenetic analysis of the 109 RpbHLH protein sequences was carried out, and the RpbHLH proteins were divided into 31 subfamilies (1–31) (Figure 3A).

The analysis of the *RpbHLH* structures revealed that the numbers of introns and exons of the 109 identified *RpbHLH*s within the same subfamily were also similar, especially among homologous branches. As indicated in Figure 3B, the *RpbHLH* family contained between 1 and 15 exons, with 20 *RpbHLHs* having 3 exons and 42 *RpbHLH*s possessing 2 to 7 exons.

The conservation motifs and gene structures of the 109 identified *RpbHLH*s were analyzed using MEME 5.4.1. As illustrated in Figure 3D, the analysis revealed six conserved motifs (motifs 1–6). Motif 1 contained an α-helix 1 region and a half-loop region, while motif 2 included an α-helix 2 region and a loop region. Motif 3 was primarily composed of an acidic region. The majority of the *RpbHLH* family contained motifs 1 and 2, with the exception of *RpbHLH35/3*, which solely contained motif 1 (Figure 3C). Furthermore, the conserved motifs of *RpbHLH*s within the same subfamily were highly similar, suggesting functional conservation within subfamilies. These findings provide valuable insights into the evolutionary and structural diversity of the *RpbHLH* family. The conserved motifs and gene structures are crucial for understanding the functional roles of these genes in regulating key processes, such as flower color and development, in *R*. *pulchrum*.

### 2.5. Chromosomal Localization and Collinearity Analysis of RpbHLHs

**Figure 4:**
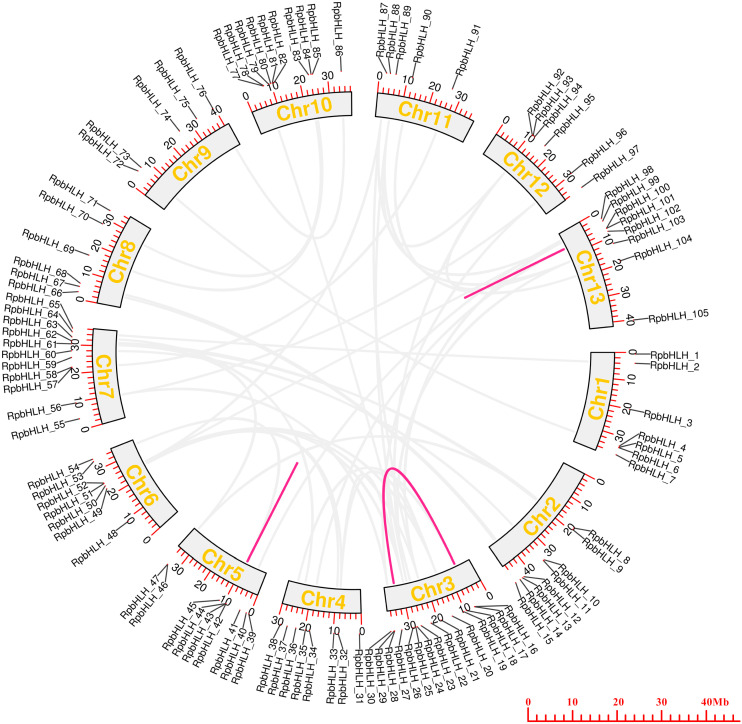
The collinearity analysis of *RpbHLH*s in the *R. pulchrum* genome. The colored outer circles represent chromosomes, with duplicated *RpbHLHs* mapped to different chromosomes, as shown in the middle section. *RpbHLHs* exhibiting collinearity relationships are linked by lines. The light gray lines represent homology between genes on different chromosomes, while the magenta lines indicate homology within the same chromosome.

To examine the chromosomal distributions of the *RpbHLH* families, each identified *RpbHLH* was mapped to 13 physical positions on the *R. pulchrum* chromosomes according to the gene annotation information (Table S2). As shown in Figure 4, on average, each chromosome contained 5–7 *RpbHLH*s. Chromosome 3 and chromosome 7 contained 15 and 11 *RpbHLH*s, respectively, while only 1 *RpbHLH* was found in chromosomes 143, 216, 228, and 235. A homology analysis of the *Rhododendron* genes was performed using OrthoFinder2 2.5.4, identifying 59 pairs of homologous genes. Among them, 56 pairs exhibited homology between gene chromosomes, while 3 pairs of genes, namely *RpbHLH42* and *RpbHLH43*, *RpbHLH42* and *RpbHLH44*, and *RpbHLH100* and *RpbHLH101*, exhibited homology within the same chromosome. The ratio of the number of non-synonymous substitutions per non-synonymous site (Ka) to the number of synonymous substitutions per synonymous site (Ks), or Ka/Ks, was calculated for the 59 pairs of homologous genes. The Ka/Ks ratio reflects a gene’s evolutionary processes and selection pressures. A Ka/Ks ratio of less than 1 for all pairs indicates that the *RpbHLH*s underwent purifying selection during evolution, which plays a critical role in maintaining the stability of the *bHLH* conserved domain (Table S3). These findings underscore the importance of the *RpbHLH* family in maintaining the genetic integrity of *R*. *pulchrum* while also suggesting potential roles in flower color regulation and development. The distribution and evolutionary patterns of these genes could help elucidate their involvement in the molecular mechanisms that govern flower pigmentation and developmental processes in *R*. *pulchrum*.

### 2.6. Prediction of the Regulatory Elements in RpbHLH Promoters

**Figure 5:**
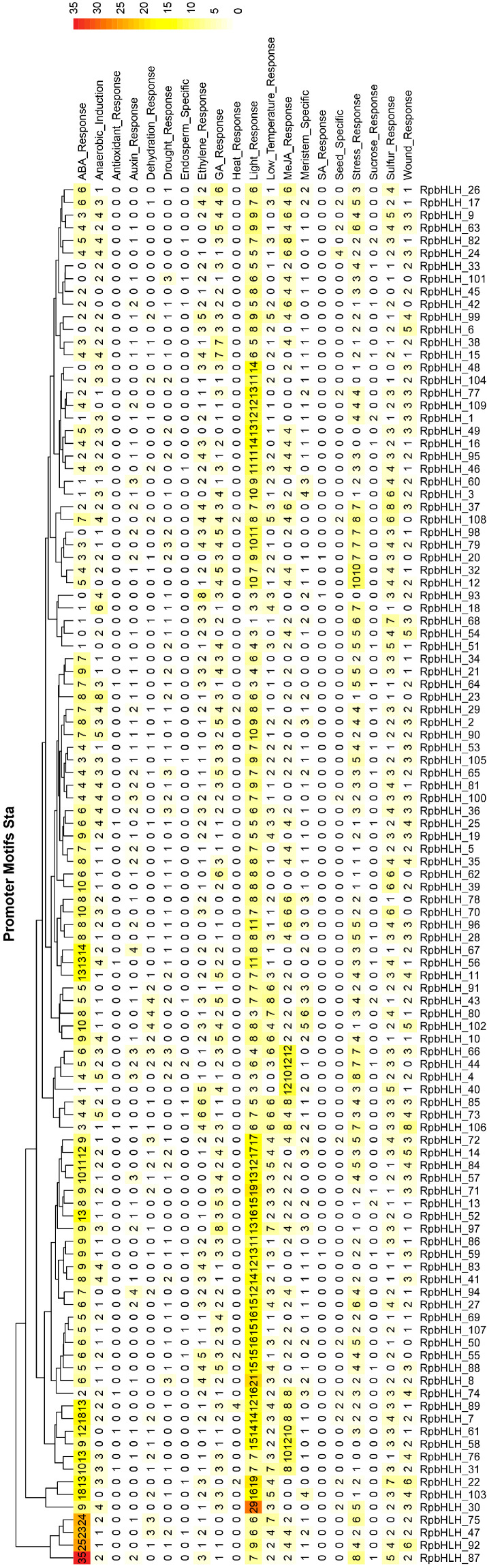
Prediction of cis-acting elements in *RpbHLH* promoter regions. The distribution of cis-acting elements in the upstream 2000 bp promoter regions of the *RpbHLH*s.

The analysis of cis-acting elements in the promoter regions of the *RpbHLH*s significantly enhanced our understanding of their regulation, function, and evolutionary dynamics (Table S4). As illustrated in Figure 5, all *RpbHLH*s contain cis-acting elements associated with light response, indicating that light signaling plays a crucial role in modulating the expression of these genes. Additionally, many *RpbHLH*s feature cis-acting elements related to various environmental factors and hormonal responses: 102 *RpbHLH*s contain GA_Response elements, 96 have Wound_Response elements, 92 include Ethylene_Response elements, 77 carry MeJA_Response elements, 60 feature Drought_Response elements, 57 contain Auxin_Response elements, and 5 include Heat_Response elements. The distribution of these cis-acting elements suggests that *RpbHLH*s play a pivotal role in regulating flower growth and development, particularly in response to environmental stresses such as light, drought, and mechanical damage. The presence of ethylene and MeJA response elements highlights the involvement of these genes in controlling flower senescence, color changes, and flowering processes. This is of particular significance for flower color formation and maintenance, as both the ethylene and jasmonate pathways are key regulators of flower opening and color transitions.

### 2.7. A KEGG Enrichment Analysis of Target RpbHLHs

**Figure 6:**
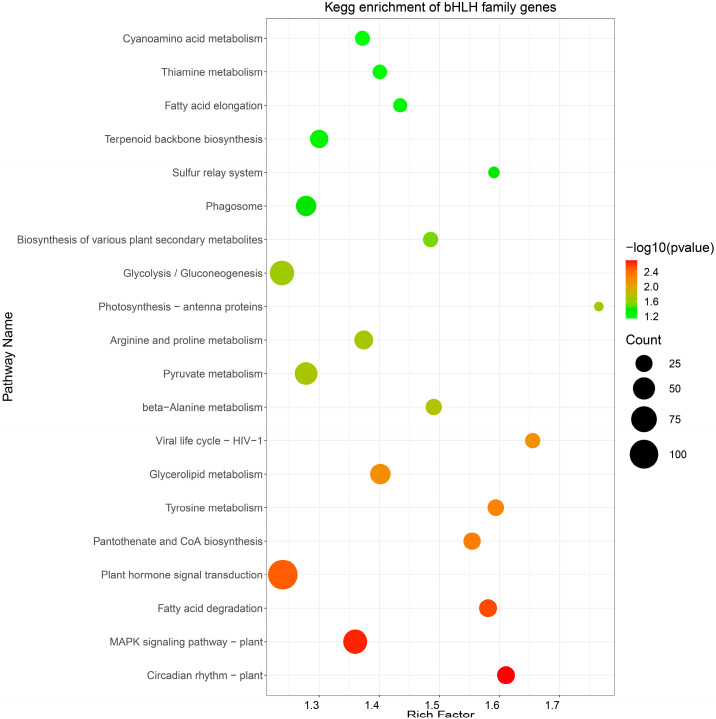
KEGG enrichment analysis. The pathway enrichment of *bHLH* genes in *R*. *pulchrum*. The size of each circle indicates the number of genes enriched in each pathway, while the color gradient represents the significance of the enrichment, with darker green indicating higher significance (lower *p*-values).

A KEGG pathway analysis was conducted to perform functional enrichment of the predicted target *RpbHLHs* (Table S5). The resulting KEGG enrichment pathway map revealed the enrichment of potential target *RpbHLHs* in 20 distinct pathways (Figure 6). Among these, the pathways exhibiting the highest number of enriched genes were plant hormone signal transduction (109 genes), glycolysis/gluconeogenesis (65 genes), and the MAPK signaling pathway—plant (62 genes). Additionally, the flavonoid biosynthesis pathway, which plays a key role in plant pigmentation, showed the enrichment of 25 genes. These metabolic and signaling pathways provide the necessary energy and regulatory signals to support floral organ development and color formation and the plant’s response to environmental stressors.

### 2.8. The Expression of RpbHLHs at Different Developmental Stages and in Flowers of Different Colors

**Figure 7:**
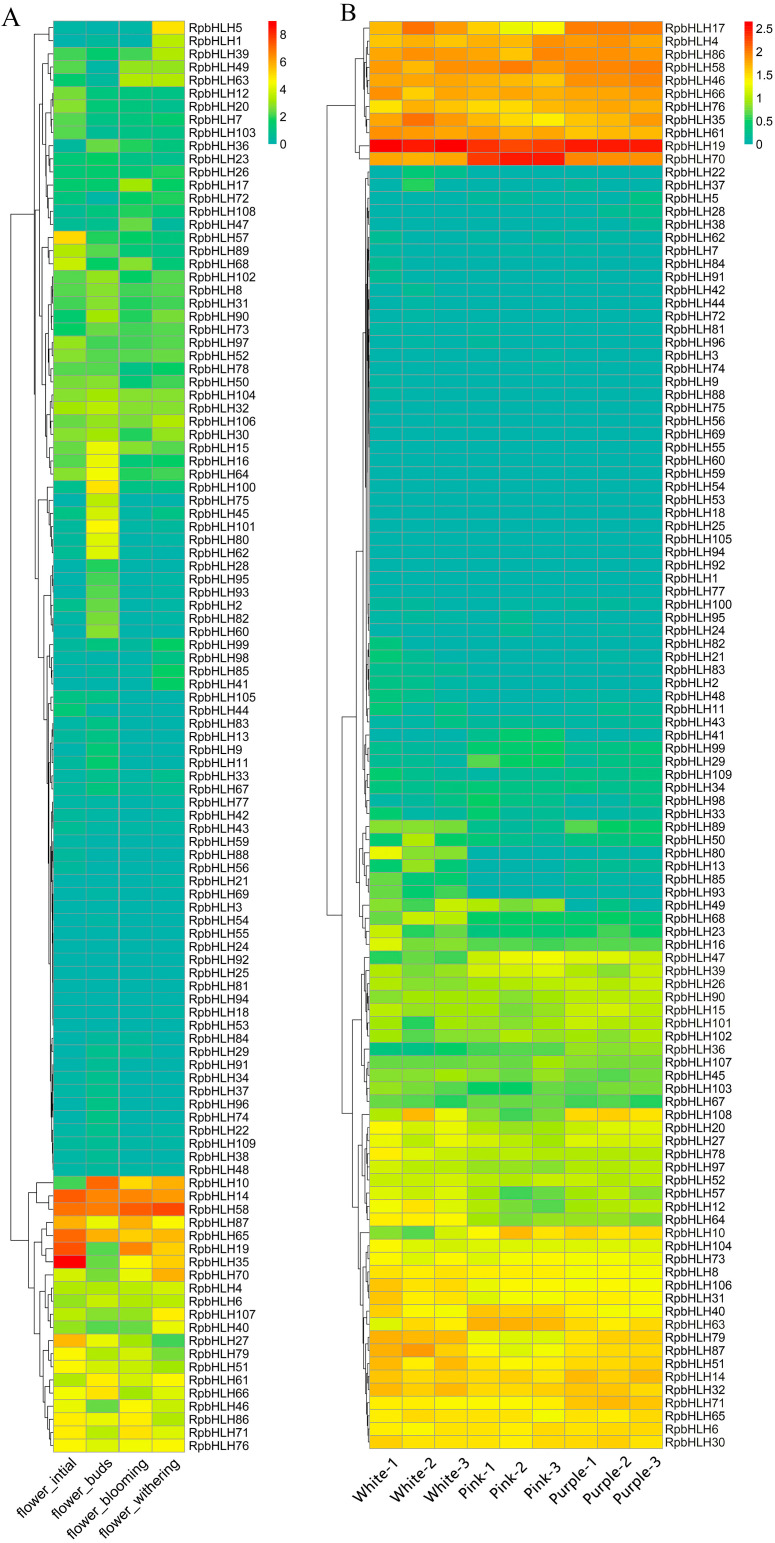
The expression of *RpbHLHs* in different growth stages and flowers of different colors. (**A**) *RpbHLH* expression in *R*. *pulchrum* flower initiation, flower budding, flower withering, and flower blooming stages. The data were sourced from the NCBI bioprojects (PRJNA485857). (**B**) *RpbHLH* expression in white, pink, and purple flowers. The data were measured in this study. The color intensity represents the expression levels, with higher expression indicated by warmer colors (yellow to red) and lower expression by cooler colors (green to blue).

To explore the potential role of the identified *RpbHLHs* in different developmental stages, the expression of the *RpbHLHs* was analyzed. As shown in Figure 7A, the expression levels of the *RpbHLH*s were higher during the bud stage compared to those during the early flowering, blooming, and fading stages. Seven genes, including *RpbHLH14*, *RpbHLH58*, *RpbHLH87*, *RpbHLH65*, *RpbHLH19*, *RpbHLH35*, and *RpbHLH27*, exhibited high expression during the bud stage. In the early flowering stage, four genes (*RpbHLH10*, *RpbHLH14*, *RpbHLH58*, and *RpbHLH65*) were highly expressed. During the blooming stage, seven genes (*RpbHLH10*, *RpbHLH14*, *RpbHLH58*, *RpbHLH65*, *RpbHLH19*, *RpbHLH35*, and *RpbHLH70*) demonstrated high expression. In the fading stage, six genes (*RpbHLH10*, *RpbHLH14*, *RpbHLH58*, *RpbHLH87*, *RpbHLH65*, and *RpbHLH19*) were highly expressed. Moreover, the expression of *RpbHLH14, RpbHLH58*, and *RpbHLH65* increased across all four stages (Figure 7A).

The expression of the *RpbHLH*s in flowers of different colors (white, pink, and purple) was also evaluated (Figure 7B). The overall RPKM (Reads Per Kilobase of transcript per Million mapped reads) values indicate that white flowers had the highest number of expressed genes, followed by purple and pink flowers (Table S6). *RpbHLH4*, *RpbHLH19*, *RpbHLH86*, *RpbHLH58*, *RpbHLH46*, *RpbHLH66*, *RpbHLH76*, *RpbHLH 35*, and *RpbHLH61* exhibited high expression levels across all three flower colors. *RpbHLH70* showed high expression in pink flowers and moderate expression in white and purple flowers, while *RpbHLH17* was exclusively expressed in the white and purple flowers. Among the 11 genes (*RpbHLH17*, *RpbHLH4*, *RpbHLH86*, *RpbHLH58*, *RpbHLH46*, *RpbHLH66*, *RpbHLH76*, *RpbHLH108*, *RpbHLH14*, *RpbHLH32,* and *RpbHLH71*) highly expressed in the purple flowers, *RpbHLH17* demonstrated the highest expression levels. In white flowers, the genes *RpbHLH35*, *RpbHLH61*, *RpbHLH79*, *RpbHLH87*, and *RpbHLH5*1 were highly expressed. Conversely, in pink flowers, only three genes (*RpbHLH22*, *RpbHLH40,* and *RpbHLH63*) were highly expressed.

Overall, the expression profiles of the *RpbHLH*s across developmental stages and flower colors highlight their complex and dynamic role in flower development, color formation, and adaptation. The genes that show consistent expression across different stages and flower colors are likely involved in fundamental processes such as flower initiation, maturation, and pigmentation. These data underscore the potential of *RpbHLH*s in regulating the intricate network of pathways that govern flower color diversity and development in *R*. *pulchrum*.

### 2.9. Quantification of the RpbHLH Expression Using RT-qPCR

**Figure 8:**
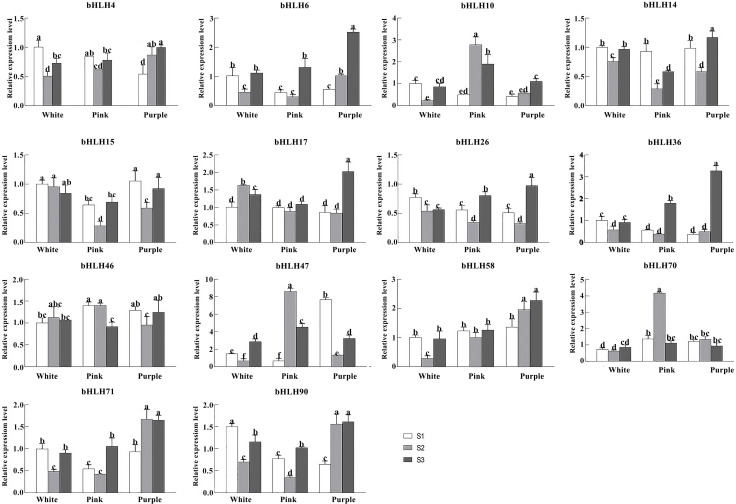
The expression of *RpbHLH*s in flowers of different colors and different stages in *Rhdodendron pulchrum*. RT-qPCR analysis of 14 genes (*RpbHLH4*, *RpbHLH6*, *RpbHLH10*, *RpbHLH14*, *RpbHLH15*, *RpbHLH17*, *RpbHLH26*, *RpbHLH36*, *RpbHLH46*, *RpbHLH47*, *RpbHLH58*, *RpbHLH70*, *RpbHLH71*, and *RpbHLH90*) across various flower colors and developmental stages (the bud stage [S1], the early flowering stage [S2], and the peak flowering stage [S3]). The experiments were independently replicated a minimum of three times. The data are presented as the mean ± standard error (*n* = 3), with distinct letters signifying statistically significant differences (*p* < 0.05) as determined by Duncan’s multiple range test.

To elucidate the influence of *RpbHLHs* on varied developmental stages and floral pigmentation in *R. pulchrum* further, highly expressed *RpbHLH*s from the transcriptome data were assessed using RT-qPCR (Table S7). The expression of 14 genes from different subfamilies in flowers of different colors (white, pink, and purple) and at different developmental stages (the bud stage (S1), the early flowering stage (S2), and the blooming stage (S3)) was quantified (Figure 8). In white flowers, *RpbHLH58*, *RpbHLH71*, *RpbHLH36*, *RpbHLH15*, *RpbHLH14*, *RpbHLH4*, *RpbHLH90*, *RpbHLH10*, and *RpbHLH26* were highly expressed in the bud stage, while *RpbHLH17* and *RpbHLH46* were highly expressed in the early flowering stage, and *RpbHLH6*, *RpbHLH70*, and *RpbHLH47* were highly expressed in the blooming stage. Eleven genes (*RpbHLH57*, *RpbHLH71*, *RpbHLH6*, *RpbHLH36*, *RpbHLH14*, *RpbHLH4*, *RpbHLH90*, *RpbHLH70*, *RpbHLH47*, *RpbHLH10*, and *RpbHLH26*) exhibited an expression pattern of initially decreasing and subsequently increasing during different developmental stages, while the expression of *RpbHLH17* and *RpbHLH46* displayed the opposite pattern; the expression of *RpbHLH15* consistently decreased during the developmental process. In pink flowers, *RpbHLH14* and *RpbHLH4* were highly expressed in the bud stage; *RpbHLH70*, *RpbHLH47*, and *RpbHLH10* were highly expressed in the early flowering stage; and *RpbHLH58*, *RpbHLH17*, *RpbHLH71*, *RpbHLH6*, *RpbHLH36, RpbHLH15*, *RpbHLH90*, and *RpbHLH26* were highly expressed in the blooming stage. Ten genes (*RpbHLH58*, *RpbHLH17*, *RpbHLH71*, *RpbHLH6*, *RpbHLH36*, *RpbHLH15*, *RpbHLH14*, *RpbHLH4*, *RpbHLH90*, and *RpbHLH26*) exhibited an expression pattern of initially decreasing and subsequently increasing during the developmental process, while *RpbHLH10* and *RpbHLH47* displayed the opposite pattern.

In purple flowers, *RpbHLH46*, *RpbHLH15*, and *RpbHLH70* exhibited high expression levels during the bud stage. *RpbHLH70* maintained high expression in the early flowering stage, while *RpbHLH58*, *RpbHLH17*, *RpbHLH6*, *RpbHLH36*, *RpbHLH14*, *RpbHLH4*, *RpbHLH90*, *RpbHLH10*, and *RpbHLH26* demonstrated elevated expression during the blooming stage. The expression levels of *RpbHLH58*, *RpbHLH71*, *RpbHLH4*, *RpbHLH90*, and *RpbHLH10* increased throughout the flowering process. *RpbHLH70* displayed an initial increase followed by a decrease in expression. In contrast, *RpbHLH46*, *RpbHLH6*, *RpbHLH36*, *RpbHLH15*, *RpbHLH14*, *RpbHLH70*, and *RpbHLH26* showed an initial decrease succeeded by an increase in expression. Overall, the data suggest that *bHLH* genes have dynamic and color-specific expression profiles that may regulate both the developmental processes and pigmentation pathways in different flower colors.

## 3. Discussion

Studies have demonstrated that the *bHLH* gene family is widely present in higher plants and plays a crucial role in plant growth, development, stress responses, and defense reactions [15]. A total of 109 *bHLH* genes (*RpbHLHs*) were identified from the *R*. *pulchrum* genome, and their physicochemical properties and subcellular localization were analyzed further. Through a phylogenetic analysis, we compared 109 *RpbHLH*s with 166 *bHLH* genes from *Arabidopsis thaliana* and constructed an evolutionary tree. The results revealed significant evolutionary divergence between *R*. *pulchrum* and *Arabidopsis*, which may be linked to the unique flower color traits of *R*. *pulchrum* [16]. These genes were grouped into 31 subfamilies, with subfamily 25 containing the highest number of genes (14), while subfamily 19 contained only a single gene (*RpbHLH*). Notably, no *RpbHLH*s were found in subfamilies 6, 8, 18, 20, 21, 22, or 29, suggesting the potential loss of these subfamilies during the evolution of *R*. *pulchrum* [8]. Some subfamilies contained only *AtbHLH* genes, while others included both *RpbHLHs* and *AtbHLH* genes, indicating species-specific differences in gene family evolution [10].

Basic helix–loop–helix (*bHLH*) transcription factors in ornamental plants exhibit highly conserved structural features that underpin their roles in floral pigmentation and morphogenesis. The results show that nearly all bHLH proteins harbor core helix–loop–helix motifs (motif 1 and motif 2) across species, reflecting a preserved domain architecture crucial for DNA binding and dimerization [17]. Gene structure is likewise conserved within *bHLH* subfamilies; members of the same clade tend to share similar exon–intron organization [18]. Notably, many anthocyanin-related *bHLHs* fall into intron-poor subgroups (with some being completely intronless), indicating an evolutionary streamlining that is consistent within these clades [18]. Such consistency in the motif composition and gene structure across diverse ornamental species suggests strong purifying selection maintaining these features over time [19,20]. The intact *bHLH* domain is almost universally maintained, ensuring that bHLH proteins can form the MYB–bHLH–WD40 (MBW) complexes that activate anthocyanin biosynthetic genes [21,22,23]. For example, transcriptomic studies in *R*. *pulchrum* have identified bHLH regulators whose high expression correlates with intense petal pigmentation, acting as positive regulators of anthocyanin accumulation [23]. Beyond pigment production, the cis-elements associated with bHLH genes are enriched in developmental and light-responsive motifs, hinting at broader roles in floral organ development and environmental response [17].

Flower colors such as red, pink, purple, and blue are largely determined by flavonoid pigments (especially anthocyanins) whose biosynthesis is tightly controlled by transcription factors [24]. A well-conserved regulatory module is the MYB-bHLH-WD40 (MBW) complex, consisting of an *R2R3-MYB* activator, a *bHLH* co-factor, and a WD40 repeat protein [25]. Extensive studies in diverse plants show that the role of *bHLH* factors in anthocyanin regulation is functionally conserved across species. In maize, the founding MBW components (C1/Lc MYB and R/B bHLHs) were first linked to pigment regulation, and similar players have since been identified in many angiosperms [24]. *R*. *pulchrum* exhibits cultivars with white, pink, light pink, and purple flowers, making it an excellent system for studying color regulation [23]. Transcriptome analyses of these differently colored petals have identified several *bHLH* genes whose expression correlates with anthocyanin accumulation. Notably, five *R*. *pulchrum bHLH* homologs (designated *RpbHLH4*, *RpbHLH14*, *RpbHLH17*, *RpbHLH58*, and *RpbHLH70*) show elevated transcript levels in pigmented pink/purple flowers compared to those in white flowers (which lack anthocyanin). In particular, one *bHLH* gene (Rhsim08G0230000, corresponding to *RpbHLH17*) was significantly upregulated in deep purple petals (light pink or white), indicating that it acts as a positive regulator of anthocyanin biosynthesis in this species [23,26].

*bHLH* transcription factors generally function as part of the conserved MYB-bHLH-WD40 complex, where they stabilize and enhance *MYB* activator binding to promoters of anthocyanin biosynthetic genes [22]. When an appropriate *bHLH* partner is present and highly expressed, the MBW complex efficiently activates enzymes in the anthocyanin pathway, leading to the accumulation of red, pink, or purple pigments in the petals [24]. Conversely, the absence or low expression of a functional *bHLH* gene can result in diminished anthocyanin production and thus lighter or white flowers [24,27]. The functional conservation of this mechanism is evident in *R*. *pulchrum*, where *bHLH* genes like *RpbHLH17*, *RpbHLH58*, and others are highly expressed in richly colored flowers, correlating with increased anthocyanin contents (Figure 1 and Figure 8).

The enrichment analysis suggests that the target *RpbHLHs* are involved in regulating a range of critical processes, such as hormonal signaling, energy metabolism, and stress responses. Plant hormones such as auxins, gibberellins, and ethylene are known to influence the synthesis of flavonoids and anthocyanins, which are key contributors to flower pigmentation [28]. The involvement of *RpbHLH*s in regulating plant hormone networks thus suggests an indirect but significant role in the control of pigment biosynthesis, further supporting their importance in flower color regulation. Furthermore, the flavonoid biosynthesis pathway, which directly influences flower color, highlights the role of RpbHLHs in regulating the synthesis of the compounds responsible for pigmentation [29]. The KEGG pathway analysis revealed that genes involved in the flavonoid biosynthesis pathway (ko00941) are crucial in regulating flower color. The flavonoid biosynthesis pathway includes essential enzymes like chalcone synthase (CHS), which are involved in the production of anthocyanins and other pigments, directly influencing flower color. Genes such as *RpbHLH17*, *RpbHLH61*, and other *bHLH* family members have been identified as similar to chalcone synthases (CHSs), confirming their role in the flavonoid biosynthesis pathway. According to the SWISS database, *RpbHLH17*/*19*/*46*/*58*/*61* are classified as chalcone synthases, which play a pivotal role in the synthesis of anthocyanins and other pigments, thus contributing to flower color regulation [30]. The *bHLH* genes *RpbHLH4*/*76*/*86* share homology with *AtbHLH11*/*34*/*47*/*104*/*105*/*115*/*121*, all belonging to the Myc-like bHLH family. This family enhances the efficiency of the amino acid residues involved in anthocyanin biosynthesis, thus playing a crucial role in flower pigmentation. Studies have shown that *AtbHLH011* (*bHLH168*) regulates anthocyanin biosynthesis in purple cabbage, and *AtbHLH034* (*EGL1*) controls pigment synthesis and accumulation in plants, thereby influencing flower color [31,32].

The *R. pulchrum* transcriptome data reveal that several *RpbHLH*s (*RpbHLH14*, *58*, *65*, *10*, *70*, and *26*) show peak expression at the floral bud, early flowering, or full-bloom stages, suggesting that they perform stage-specific regulatory functions in flower development. The enrichment of genes in the plant hormone signal transduction pathway suggested that the RpbHLHs were closely involved in regulating plant hormone networks. This is particularly relevant to flower development, as plant hormones such as auxins, gibberellins, ethylene, and jasmonic acid regulate various aspects of flower growth, maturation, and pigmentation [33]. For instance, *RpbHLH10* and *RpbHLH26* are highly expressed in floral buds and early flowers, which might relate to initiating pollen and anther development during early floral bud formation. The high bud-stage expression of *RpbHLH14* and *RpbHLH58* points to their roles in floral initiation and growth, possibly analogous to *bHLH* factors in hormone signaling (such as *MYC2*/*JAM* family members) that activate JA- or auxin-responsive genes to promote bud outgrowth and stamen maturation [34]. By full bloom, *RpbHLH65* and *RpbHLH70* are strongly upregulated, which may reflect functions in flower opening and organ expansion—comparable to *bHLH* genes like *BIGPETAL* that constrain petal size or those integrating GA and light signals into timing anthesis [35,36]. Integrating these findings on the *R*. *pulchrum* transcriptome with the cross-species literature suggests that these *bHLH* transcription factors likely coordinate flower development by controlling meristem identity, organ identity and growth, and the timing of flowering events via hormonal and developmental pathways [35,37]. Other enriched pathways, such as glycolysis/gluconeogenesis and the MAPK signaling pathway, indicate that *RpbHLHs* also play a role in managing energy metabolism and stress responses, which are essential for optimal flowering.

This study focused on identifying the *RpbHLH* family in *R. pulchrum* and analyzing its potential roles in flower color regulation. Flower color metabolite identification was not included in this study due to time and resource constraints. However, future research will aim to use metabolomics or chromatography to identify and quantify the specific compounds involved in flower color formation, providing a more comprehensive understanding of how these genes contribute to pigmentation.

## 4. Materials and Methods

### 4.1. Plant Materials

In this study, 45 healthy and stable potted *R. pulchrum* (*Rhododendron* × *pulchrum* Sweet) plants were selected from two-year-old specimens and raised in an artificial climate incubator. The plants were transferred into incubator conditions two weeks prior to sampling to acclimate. The artificial climate incubator maintained a photoperiod of 16 h of light/8 h of dark, with a light intensity of 600 μmol m^−2^ s^−1^, temperatures of 22 °C during the day and 18 °C at night, and a humidity level of 70%. The plants were categorized into three groups based on flower color: white, pink, and purple. All three groups belonged to the same variety and were grown under identical cultivation conditions.

Although the plants belong to the same variety, variations in flower color may arise from genetic differences, environmental influences, or developmental stages. To minimize potential environmental effects, all plants were maintained under identical conditions in the climate incubator. Each flower color group was further subdivided into three biological replicates, each containing five plants. Samples were collected at three distinct developmental stages: the bud stage (S1) on 23 March 2023; the early flowering stage (S2) on 15 April, just prior to full bloom; and the blooming stage (S3) on 25 April, when the flowers were fully open. At each time point, three biological replicates, each weighing approximately 0.1 g, were randomly selected from the five plants and immediately frozen in liquid nitrogen for storage at −80 °C. In total, 27 samples were collected and categorized into different groups: the white flower bud stage (whiteS1-1, whiteS1-2, whiteS1-3), the early white flowering stage (whiteS2-1, whiteS2-2, whiteS2-3), and the white flower blooming stage (whiteS3-1, whiteS3-2, whiteS3-3); the pink flower bud stage (pinkS1-1, pinkS1-2, pinkS1-3), the early pink flowering stage (pinkS2-1, pinkS2-2, pinkS2-3), and the pink flower blooming stage (pinkS3-1, pinkS3-2, pinkS3-3); and the purple flower bud stage (purpleS1-1, purpleS1-2, purpleS1-3), the early purple flowering stage (purpleS2-1, purpleS2-2, purpleS2-3), and the purple flower blooming stage (purpleS3-1, purpleS3-2, purpleS3-3) (Figure 1).

### 4.2. Determination of Flavonoids and Anthocyanins

The contents of flavonoids and anthocyanins were determined using ultraviolet–visible spectrophotometry [38,39], using a Spark instrument (Tecan Austria GmbH, Grödig, Austria).

### 4.3. RNA Extraction, cDNA Synthesis, and Quantitative Real-Time PCR Analysis

Total RNA was extracted from the plant samples using the RNAprep Pure Kit (Tiangen, Hangzhou, China). To remove trace amounts of DNA, the RNA samples were treated with RNase-free DNase I. The concentration and purity of the RNA were assessed using a micro-spectrophotometer. For the optimal results, the RNA concentration should exceed 200 ng/μL. To measure the concentration, 1 μL of RNA was analyzed using a micro-spectrophotometer. Typically, a concentration above 100 ng/μL is desired, and gel electrophoresis of the RNA should reveal two distinct bands, representing the 28S and 18S ribosomal RNA subunits (Figure S1).

The extracted RNA was utilized as the template to generate cDNA using the Evo M-MLV One Step RT-PCR Kit (AG11606, ACCURATE BIOTECHNOLOGY (HUNAN), Changsha, China) following the manufacturer’s instructions. The reaction system consisted of 2 µL of gDNA Clean Reaction Mix Ver.2, 4 µL of 5× Evo M-MLV RT Reaction Mix Ver.2, 2 µL of RNA, and RNase Free ddH_2_O to a total volume of 20 µL. The reaction conditions were set at 37 °C for 15 min and 85 °C for 5 s and then maintained at 4 °C. The resulting cDNA was stored at −20 °C for subsequent use. The primers for quantitative reverse transcription polymerase chain reaction (qRT-PCR) were designed using Primer3 (https://primer3.ut.ee, accessed on 28 June 2024) and synthesized by Qingke Biotechnology Co., Ltd. (Hangzhou, China). *RpUBQ* was selected as the internal reference gene [40]. For qRT-PCR, 10-fold-diluted cDNA was employed as the template, and the reaction was performed using the SYBR Green Premix Pro Taq HS qPCR Kit (AG11701, ACCURATE BIOTECHNOLOGY (HUNAN), Changsha, China) and analyzed using the LightCycler^®^480 II (Roche Diagnostics, Mannheim, Germany). The reaction system of 10 µL included 5 µL of 2× SYBR Green Pro Taq HS Premix, 1 µL of the cDNA template, 0.2 µL of the primer (at a final concentration of 0.2 mM), and 3.6 µL of RNase Free ddH_2_O (Table S8). The reaction program consisted of pre-denaturation at 95 °C for 30 s, denaturation at 95 °C for 5 s, and annealing and extension at 60 °C for 30 s, with a total of 40 cycles. Three biological replicates were set for each sample, and the relative expressions of the candidate genes were calculated using the 2^−ΔΔCt^ method [41].

All experimental data were analyzed using a one-way ANOVA in SPSS 19.0 software (IBM Corporation, Armonk, NY, USA). Before conducting the ANOVA, the assumption of normality was assessed using the Shapiro–Wilk test. When *p* > 0.05, the data were considered to follow a normal distribution. The assumption of homogeneity of variances was tested using Levene’s test, and when *p* > 0.05, the variances among the treatment groups were considered equal. Duncan’s test was employed to determine significant differences among the samples at a threshold of *p* < 0.05. The data were then visualized using GraphPad Prism 9.0.0 (GraphPad Software, San Diego, CA, USA). To ensure reliability, each sample was tested with three biological replicates.

### 4.4. Identification and Characterization of the Physicochemical Properties of the RpbHLHs

The *RpbHLH* gene sequences were downloaded from the *R. pulchrum* genomic library (https://figshare.com, accessed on 15 March 2024) [42]. The bHLH domain PF00010.26 was obtained from the PFAM database (https://pfam.xfam.org, accessed on 18 March 2024). The presence of the bHLH conserved domain in the *R. pulchrum* proteome was identified using hmmsearch (http://hmmer.janelia.org, accessed on 18 March 2024). Subsequently, the *R. pulchrum* protein sequence was compared to the PFAM database [43] using hmmscan [44] to determine whether the protein sequence contained the *bHLH* domain. The number of amino acids was counted using the SeqKit 2.10.0 (https://github.com, accessed on 5 April 2024). The ExPASy-ProtParam tool (https://web.expasy.org, accessed on 6 April 2024) was employed to analyze the molecular weight (MW) and isoelectric point (PI) of the protein. Finally, subcellular localization predictions were performed using WoLF PSORT 1.0 [45] (https://wolfpsort.hgc.jp, accessed on 7 April 2024).

### 4.5. Chromosomal Localization, Collinearity Analysis, and Ka/Ks Calculation of RpbHLHs

Homologous genes were identified using OrthoFinder 2.5.2 (https://github.com, accessed on 9 April 2024) [46], and TBtools 2.310 [47] (https://github.com, accessed on 12 April 2024) was used to draw a collinearity map of these genes on the chromosome. The synonymous substitution rate (Ks) and the non-synonymous substitution rate (Ka) of homologous genes were calculated using KaKs_Calculator 2.0 (https://ngdc.cncb.ac.cn, accessed on 15 April 2024) [48].

### 4.6. Prediction of the Promoter’s Regulatory Elements

To predict the regulatory elements of the *RpbHLH* promoter, a 2000-base-pair (bp) upstream region of the *RpbHLH* starting codon was extracted and defined as the promoter region. The PlantCARE database [49] (http://www.biodata-tech.com, accessed on 20 April 2024) and the plant cis-acting regulatory DNA elements (PLACE) database [50] (https://www.dna.affrc.go.jp, accessed on 21 April 2024) were utilized to predict regulatory elements within the promoter sequence. A physical map illustrating the positions of these elements was generated, and the frequency of each element was quantified.

### 4.7. Multiple Gene Alignment and Phylogenetic Analysis of the RpbHLHs

The RpbHLH proteins were aligned with *Arabidopsis* AtbHLH proteins. The *Arabidopsis* protein sequences were obtained from The Arabidopsis Information Resource (TAIR) (http://www.arabidopsis.org, accessed on 7 May 2024). The alignment was performed using the default parameters in MAFFT 7.313 [51]. The maximum likelihood (ML) phylogenetic tree of the *RpbHLH* family was constructed using FastTree [52] using the general time-reversible (GTR) model and the Gamma20 model of rate heterogeneity. The phylogenetic tree was assessed using the Shimodaira–Hasegawa test.

### 4.8. Structure and Motif Analyses of RpbHLHs

The gene structures of the *RpbHLHs* were visualized using the Gene Annotation GFF library [42] (https://figshare.com, accessed on 11 May 2024). The motifs in the RpbHLH family proteins were analyzed using the MEME software [53] (http://meme-suite.org, accessed on 12 May 2024), with the parameter for identifying the motifs set to 6, while the other parameters were maintained at their default values. The motif structure and gene structure diagrams were generated based on the motif prediction results and reference genome annotation data.

### 4.9. Prediction and Enrichment Analysis of Target Genes of RpbHLHs

PlantRegMap [54] was employed to predict the target genes of the RpbHLHs by identifying bHLH binding sites within the 2 kb promoter region of the entire genome, utilizing a stringent significance threshold of *p* ≤ 1 × 10^−8^. Following the identification of the target genes, a Kyoto Encyclopedia of Genes and Genomes (KEGG) enrichment analysis was conducted to ascertain significant biological pathways.

### 4.10. The Spatiotemporal-Specific Expression Analysis of the RpbHLHs

Total RNA was extracted from flowers of different colors using an RNAprep Pure Plant Plus kit (DP441, Tiangen Biotech Co., Ltd., Beijing, China). The RNA library was prepared using an Illumina (San Diego, CA, USA) TruSeq RNA kit 2.0 and subsequently sequenced on the Illumina NovaSeq 6000 platform in PE150 mode according to Li et al. [55]. The RNA-seq data were deposited into the NCBI Sequence Read Archive (accession number: PRJNA1265659). The RNA-seq data for various developmental stages were obtained from the NCBI bioprojects PRJNA485857. The RPKM values of these *RpbHLH*s were calculated in transcripts per million (TPM), and heatmaps of the results were generated using R 3.5.2, with the colors representing log2^(TMP+1)^.

## 5. Conclusions

This study highlights the significant role of the *bHLH* gene family in *R*. *pulchrum*, particularly in regulating flower color and development. We identified 109 *RpbHLH*s, which exhibited diverse characteristics in terms of the amino acid count, molecular weight, and isoelectric point. The evolutionary analysis revealed notable differences between the *RpbHLH*s in *R*. *pulchrum* and *Arabidopsis thaliana*, with some genes specific to *R*. *pulchrum*, suggesting their role in flower color formation and development. A further analysis of the gene structure showed variability in the number of exons, with conserved motifs across different subfamilies, indicating functional conservation. The presence of conserved motifs provides insight into the regulatory roles of these genes in flower color and development. *RpbHLH*s are distributed across several chromosomes and respond to environmental factors like light, mechanical stress, and drought. The promoter analysis further indicates these genes are involved in both flower color regulation and hormone signaling pathways. The expression analysis showed significant differences in the *RpbHLH* expression across different flower colors (white, pink, and purple) and developmental stages (bud, early flowering, and blooming). For example, genes like *RpbHLH14*, *RpbHLH58*, and *RpbHLH65* were highly expressed in white flowers, particularly during the bud and blooming stages. In purple flowers, genes such as *RpbHLH17* and *RpbHLH58* showed significantly higher expression. The KEGG pathway analysis revealed that *RpbHLH*s are involved in multiple important pathways, such as plant hormone signaling, glycolysis/gluconeogenesis, and MAPK signaling, highlighting their role in flower color regulation and development, especially in flavonoid biosynthesis, which is closely linked to flower pigmentation. Collectively, these findings enhance our understanding of the structure of the *RpbHLH* family and provide a foundation for further investigation into the regulatory function of *bHLH* genes in flower color and development in *R. pulchrum* splendida.

## Data Availability

The RNA-seq data in this study were deposited into the NCBI Sequence Read Archive under accession number PRJNA1265659. The other datasets generated during and/or analyzed during the current study can be made available by the corresponding author on reasonable request.

## Supplementary Materials

The following supporting information can be downloaded at https://www.mdpi.com/article/10.3390/plants14111713/s1. Figure S1: The electrophoresis gel imaging of the 28s and 18s ribosomal RNA subunits. Table S1: The analysis of the physicochemical properties of proteins in the *RpbHLH* family. Table S2: *RpbHLH* gene and protein sequence data. Table S3: *RpbHLH* pair KaKs ratio analysis. Table S4: Prediction of the cis-acting elements in the *RpbHLH* promoter region. Table S5: PlantRegMap-predicted target gene IDs. Table S6: Flower color and *RpbHLH* expression (FPKM). Table S7: ANOVA results for the *RpbHLH*s. Table S8: qRT-PCR primer sequences for *bHLH* genes.
