# Supplementary material for: Identification and Expression Analysis of the bHLH Gene Family in Rhododendron × pulchrum Sweet with Different Flower Colors
Source: Plants (Basel). 2025 Jun 4;14(11):1713. doi: 10.3390/plants14111713 (PMC12157016; doi:10.3390/plants14111713)
Supplement: Supplementary file 1 [file plants-14-01713-s001.zip › Figure S1 The electrophoresis gel imaging of the 28s and 18s ribosomal RNA subunits.pdf]

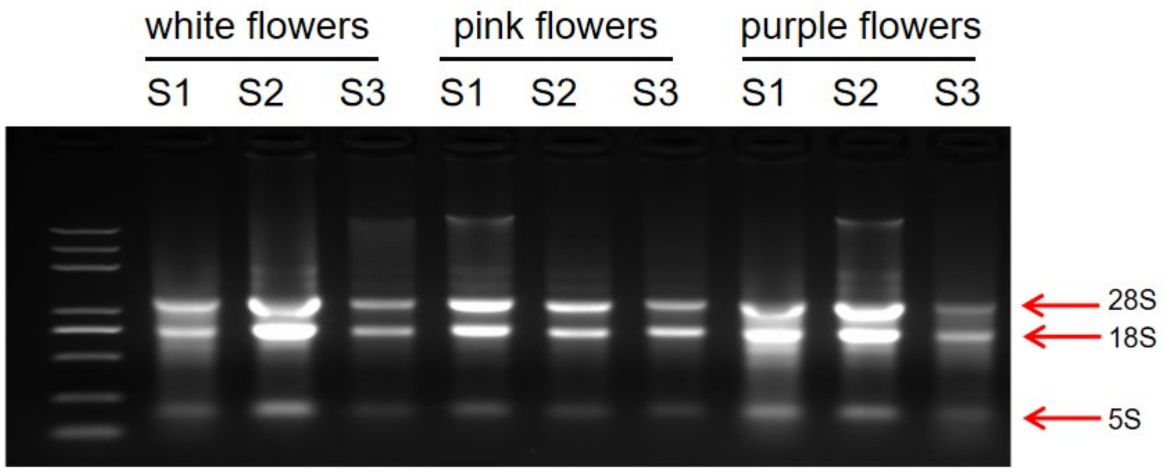


**Figure S1.** The electrophoresis gel imaging of the 28s and 18s ribosomal RNA subunits. R. pulchrum in the flower bud stage (S1), flowering stage (S2), and flower blooming stage (S3).
