# Supplementary material for: Identification and Expression Analysis of the bHLH Gene Family in Rhododendron × pulchrum Sweet with Different Flower Colors
Source: Plants (Basel). 2025 Jun 4;14(11):1713. doi: 10.3390/plants14111713 (PMC12157016; doi:10.3390/plants14111713)
Supplement: Supplementary file 1 [file plants-14-01713-s001.zip › Table S1. Analysis of the physicochemical properties of proteins in the RpbHLH gene family.pdf]

Table S1. Analysis of the physicochemical properties of proteins in the *RpbHLH* gene family

Sequence ID	amino acid count	Molecular Weight	Theoretical pI	coding sequence length	grand Average of Hydropathicity	Aliphatic Index	subcellular localization
RpbHLH 1	172	19226.27	8.86	519	-0.181	38.05	cyto
RpbHLH 2	593	66665.37	6.35	1782	-0.428	57.13	nucl
RpbHLH 3	256	29184.03	5.74	771	-0.468	55.74	nucl
RpbHLH 4	222	25202.58	5.13	669	-0.688	60.15	nucl
RpbHLH 5	349	40365.11	6.4	1050	-0.893	60.65	nucl
RpbHLH 6	293	31769.95	5.52	882	-0.355	56.7	golg
RpbHLH 7	179	19945.76	9.6	540	-0.374	46.68	cyto
RpbHLH 8	390	42787.9	6.05	1173	-0.812	44.83	nucl
RpbHLH 9	384	43044.29	5.47	1155	-0.532	44.56	nucl
RpbHLH 10	449	49147.68	5.35	1350	-0.514	75.77	nucl
RpbHLH 11	506	55984.96	6.41	1521	-0.493	56.39	vacu
RpbHLH 12	527	57331.94	8.41	1584	-0.591	64.97	nucl
RpbHLH 13	592	64365.84	6.09	1779	-0.547	60.07	nucl
RpbHLH 14	491	54037.67	6.31	1476	-0.5	76.21	nucl
RpbHLH 15	304	33977.99	6.09	915	-0.599	70	nucl
RpbHLH 16	511	56448.12	5.24	1536	-0.596	72.7	nucl
RpbHLH 17	385	43132.12	9.27	1158	-0.608	68.39	nucl
RpbHLH 18	186	20729.64	8.57	561	-0.51	89.62	cyto
RpbHLH 19	275	30326.7	5.92	828	-0.76	66.65	nucl
RpbHLH 20	618	67437.93	6.86	1857	-0.776	67.22	nucl

Continued Table S1. Analysis of the physicochemical properties of proteins in the *RpbHLH* gene family

Sequence ID	amino acid count	Molecular Weight	Theoretical pI	coding sequence length	grand Average of Hydropathicity	Aliphatic Index	subcellular localization
RpbHLH 21	279	31324.22	7.09	840	-0.603	63.94	nucl
RpbHLH 22	440	50093.67	5.67	1323	-0.456	75.57	chlo
RpbHLH 23	691	75933.45	5.06	2076	-0.277	84.24	chlo
RpbHLH 24	342	37110.73	5.23	1029	-0.327	86.14	nucl
RpbHLH 25	299	33579.07	5.75	900	-0.926	61.64	nucl
RpbHLH 26	350	38582.59	5.57	1053	-0.793	64.97	nucl
RpbHLH 27	591	64913.51	5.67	1776	-0.564	73.08	nucl
RpbHLH 28	202	21868.84	9.66	609	-0.561	75.45	nucl
RpbHLH 29	276	31669.9	8.58	831	-0.537	79.06	nucl
RpbHLH 30	699	77094.82	4.84	2100	-0.395	78.07	nucl
RpbHLH 31	249	27119.23	5.8	750	-0.671	63.57	nucl
RpbHLH 32	321	35470.11	5.78	966	-0.917	61.37	nucl
RpbHLH 33	333	37583	5.53	1002	-0.752	77.33	nucl
RpbHLH 34	764	85495.26	7.16	2295	-0.457	78.73	nucl
RpbHLH 35	206	23005.26	5.75	621	-1.051	54.51	nucl
RpbHLH 36	433	47782.73	9.11	1302	-0.674	62.4	nucl
RpbHLH 37	309	34169.53	8.97	930	-0.681	68.54	nucl
RpbHLH 38	368	40603.09	8.66	1107	-0.539	69.95	nucl
RpbHLH 39	246	28080.78	5.31	741	-0.593	79.27	nucl
RpbHLH 40	442	45827.89	6.03	1329	-0.405	67.9	nucl

Continued Table S1. Analysis of the physicochemical properties of proteins in the *RpbHLH* gene family

Sequence ID	amino acid count	Molecular Weight	Theoretical pI	coding sequence length	grand Average of Hydropathicity	Aliphatic Index	subcellular localization
RpbHLH 41	543	61050.72	6.37	1632	-0.446	77.02	nucl
RpbHLH 42	200	22837	8.99	603	-0.687	74.6	nucl
RpbHLH 43	474	52232.12	6.41	1425	-0.458	88.57	nucl
RpbHLH 44	250	27459.82	8.85	753	-0.587	76.8	nucl
RpbHLH 45	240	26227.85	8.88	723	-0.338	78.38	nucl
RpbHLH 46	768	85000.79	6.31	2307	-0.372	83.65	nucl
RpbHLH 47	344	38649.8	8.5	1035	-0.839	60.67	nucl
RpbHLH 48	408	45539.95	5.36	1227	-0.622	76.72	nucl
RpbHLH 49	280	30739.11	5.64	843	-0.665	58.18	nucl
RpbHLH 50	307	34195.44	5.97	924	-0.548	79.77	nucl
RpbHLH 51	544	58794.45	5.19	1635	-0.595	62.74	nucl
RpbHLH 52	329	36327.12	5.47	990	-0.739	73.5	nucl
RpbHLH 53	227	25749.25	5.61	684	-0.421	98.68	nucl
RpbHLH 54	343	37612.61	5.1	1032	-0.366	88.4	nucl
RpbHLH 55	330	36798.35	4.8	993	-0.864	64.18	nucl
RpbHLH 56	320	36233.4	5.15	963	-0.648	70.38	nucl
RpbHLH 57	293	32761.82	8.75	882	-0.27	91.91	nucl
RpbHLH 58	635	69753.84	5.76	1908	-0.631	65.26	nucl
RpbHLH 59	231	26357.12	5.9	696	-0.444	82.6	nucl
RpbHLH 60	429	47649.05	7.19	1290	-0.348	87.46	nucl

Continued Table S1. Analysis of the physicochemical properties of proteins in the *RpbHLH* gene family.

Sequence ID	amino acid count	Molecular Weight	Theoretical pI	coding sequence length	grand Average of Hydropathicity	Aliphatic Index	subcellular localization
RpbHLH 61	406	44698.02	6.81	1221	-0.88	46.87	nucl
RpbHLH 62	470	52320.16	5.47	1413	-0.6	73.83	nucl
RpbHLH 63	487	52463.53	8.93	1464	-0.47	62.18	nucl
RpbHLH 64	633	71012.35	5.05	1902	-0.476	81.45	nucl
RpbHLH 65	589	65283.35	6.73	1770	-0.543	69.88	nucl
RpbHLH 66	887	97247.54	5.27	2664	-0.478	78.83	nucl
RpbHLH 67	539	59077.78	5.29	1620	-0.621	61.91	nucl
RpbHLH 68	432	47606.25	7.05	1299	-0.754	56.85	nucl
RpbHLH 69	310	34459.15	5.03	933	-0.389	78.74	nucl
RpbHLH 70	265	28949.53	5.92	798	-0.403	85.7	nucl
RpbHLH 71	427	46959.12	8.23	1284	-0.708	62.86	nucl
RpbHLH 72	195	21763.19	8.28	588	-0.116	103.9	cyto_nucl
RpbHLH 73	294	31649.38	5.16	885	-0.426	69.12	nucl
RpbHLH 74	265	29988.96	5.49	798	-0.394	94.53	nucl
RpbHLH 75	505	55619.77	4.91	1518	-0.306	75.82	nucl
RpbHLH 76	237	26162.72	8.77	714	-0.647	70.51	nucl
RpbHLH 77	246	26417.17	9.47	741	-0.328	69.39	nucl
RpbHLH 78	714	79680.22	6.01	2145	-0.666	70.29	nucl
RpbHLH 79	371	40185.4	5.58	1116	-0.774	60.19	nucl
RpbHLH 80	321	36385.27	4.76	966	-0.453	72.83	nucl

Continued Table S1. Analysis of the physicochemical properties of proteins in the *RpbHLH* gene family

Sequence ID	amino acid count	Molecular Weight	Theoretical pI	coding sequence length	grand Average of Hydropathicity	Aliphatic Index	subcellular localization
RpbHLH 81	256	28208.52	9.62	771	-0.53	74.26	nucl
RpbHLH 82	307	34282.7	6.34	924	-0.466	76.22	nucl
RpbHLH 83	252	28433.26	7.05	759	-0.446	83.21	nucl
RpbHLH 84	210	23895.1	4.84	633	-0.475	81.33	nucl
RpbHLH 85	210	23834.2	6.37	633	-0.412	84.48	nucl
RpbHLH 86	225	24902.4	7.71	678	-0.627	71.96	nucl
RpbHLH 87	428	47753.05	5.14	1285	-0.671	64.28	nucl
RpbHLH 88	296	32680.87	9.27	891	-0.609	70.81	nucl
RpbHLH 89	325	36433.97	6.8	978	-0.57	74.06	nucl
RpbHLH 90	728	78285.12	5.81	2187	-0.368	69.26	nucl
RpbHLH 91	311	34801.18	8.95	936	-0.307	90.29	nucl
RpbHLH 92	254	28226.15	7.05	765	-0.898	63.39	nucl
RpbHLH 93	335	37508.43	6.04	1008	-0.545	74.51	nucl
RpbHLH 94	341	37720.7	6.03	1026	-0.435	81.99	nucl
RpbHLH 95	268	30365.28	9.74	805	-0.346	79.29	nucl
RpbHLH 96	285	31855.16	7.16	858	-0.443	81.09	pero
RpbHLH 97	291	32439.05	5.63	876	-1.01	58.32	nucl
RpbHLH 98	498	55311.69	6.44	1497	-0.35	83.03	nucl
RpbHLH 99	1406	160329.75	5.79	4221	-0.687	74.2	cyto
RpbHLH 100	556	62399.46	5.01	1671	-0.602	73.94	nucl

Continued Table S1. Analysis of the physicochemical properties of proteins in the *RpbHLH* gene family

Sequence ID	amino acid count	Molecular Weight	Theoretical pI	coding sequence length	grand Average of Hydropathicity	Aliphatic Index	subcellular localization
RpbHLH 101	531	58904.95	4.77	1596	-0.428	77.83	nucl
RpbHLH 102	365	40573.28	5.13	1098	-0.729	69.18	E.R.
RpbHLH 103	402	44769.69	6.77	1209	-0.432	76.39	nucl
RpbHLH 104	686	73855.22	5.71	2061	-0.579	62	nucl
RpbHLH 105	162	18228.55	6.58	489	-0.523	67.35	nucl
RpbHLH 106	281	29846.55	6.08	846	-0.392	70.89	nucl
RpbHLH 107	225	24903.39	6.76	678	-0.627	71.96	nucl
RpbHLH 108	372	40950.32	9.09	1119	-0.547	70.83	nucl
RpbHLH 109	714	79596.5	6.99	2143	-0.446	79.19	nucl
