# Supplementary material for: Identification and Expression Analysis of the bHLH Gene Family in Rhododendron × pulchrum Sweet with Different Flower Colors
Source: Plants (Basel). 2025 Jun 4;14(11):1713. doi: 10.3390/plants14111713 (PMC12157016; doi:10.3390/plants14111713)
Supplement: Supplementary file 1 [file plants-14-01713-s001.zip › Table S6 Flower Color and RpbHLH Gene Expression (FPKM).pdf]

Table S6. Flower Color and *RpbHLH* Gene Expression (FPKM)

	White_1	White_2	White_3	Pink_1	Pink_2	Pink_3	Purple_1	Purple_2	Purple_3
RpbHLH19	405.363223137563	302.676502550432	450.059029893313	225.058455230263	176.334201608633	194.0045085	277.226269211322	301.469325382272	304.063260129476
RpbHLH70	60.6356308011124	49.1214728835462	58.1425877348017	208.035010611895	292.611529881034	276.531316538809	81.2550728279047	78.4613586323862	72.5754913194598
RpbHLH58	69.7979719808146	48.2627713960314	77.8788817144514	77.1474067654888	96.2538464153564	67.5573348078665	86.396307786156	89.6887043291368	101.674155291885
RpbHLH86	62.2040871495475	80.1835178574037	65.5819232598666	61.3158646874655	41.8910527640058	81.5923368150298	74.9555018396928	79.3602436493038	70.5416648665546
RpbHLH17	46.2666672758853	109.931044126787	70.1751185283798	34.9442227993491	17.1815944903319	22.343879125531	96.4997284609793	92.031451827191	97.8327650688053
RpbHLH46	57.6945981975909	56.516972469825	59.6706089727837	51.5114344546537	47.0650154198116	42.8468034832218	73.0890748142491	79.7972447094428	81.6670894951528
RpbHLH66	77.7019343343543	35.7244272570179	60.6077172538725	53.4885812757574	58.1660471570412	52.8372404877195	55.6799058698391	62.0501728579609	63.9394437412808
RpbHLH61	72.4938693982304	69.7340118331085	62.7870653932468	63.6764328550234	55.3063321894159	56.7852600672232	39.5812138394215	43.3859048781105	47.1948731430393
RpbHLH4	40.0970801218438	52.25372059	40.456464260912	51.2842062034586	48.5353478714508	73.4238780204793	68.4100414285744	74.8310854603589	61.0762355947023
RpbHLH35	60.4984601314481	108.4461452	63.5503746444464	45.0005522954845	33.3492579874483	25.0643408578289	42.4077393728948	48.0117814497694	67.3163977455858
RpbHLH76	28.3566131958695	54.0904627634728	42.9484632185739	31.0012956990275	30.9736738387926	47.9403730082188	52.8338983261445	57.781620698269	49.1968316507262
RpbHLH32	44.8694110230042	35.5229126070177	46.1675682594368	33.3685142021326	31.6988100156205	35.643445765354	41.924716858802	38.3296713700319	32.8317698826128
RpbHLH14	40.6564637466357	32.2173488018034	36.5836193852805	29.6650736930977	37.3327222744943	26.7514401395168	44.5092567145939	36.2661879504583	44.3516891396699
RpbHLH63	15.6270162115545	30.4184159782555	25.2264824140031	51.6897372166189	49.3597818071103	45.8410091844111	29.9970872007463	24.0598879628858	29.8432006655156
RpbHLH87	49.0316987468159	69.1613543463156	35.2771745252615	20.7245772731169	13.9293862948019	18.3527363196695	28.6366633291482	30.6489904030662	33.4920360719375
RpbHLH51	44.3919572287126	24.8002488723468	44.890823314195	26.3148383606722	22.1213102310844	24.232346891072	31.4307278960408	32.8666099384649	35.7328871634037

Continued Table S6. Flower Color and *RpbHLH* Gene Expression (FPKM)

	White_1	White_2	White_3	Pink_1	Pink_2	Pink_3	Purple_1	Purple_2	Purple_3
RpbHLH79	49.1213021181505	46.1224913687892	48.7431927580224	17.4168737715017	13.7853845229221	14.9131253051242	28.1260920091067	34.6689724189614	34.5969188010328
RpbHLH71	25.6165295158206	25.974474557473	20.7155219634408	22.1952617630245	21.7744162256833	25.5039327080482	43.0715988443863	44.8212571771619	42.9574559211755
RpbHLH30	33.9009964835528	28.8560468739178	27.014583364483	26.6804901109756	23.9149911117849	30.4176004488368	32.266558887182	35.7915867639563	28.7882782233857
RpbHLH6	24.6127797878537	21.1287521926884	28.2677359362002	28.1548969718996	25.7448007269722	31.6277039024976	28.4866328710865	31.6816233216359	32.5026293324112
RpbHLH40	33.6588476310809	21.4685358155583	17.8070255046487	38.8797711424048	34.0601209197187	34.5776363075756	23.4576297845335	18.9388066035861	19.9323207912738
RpbHLH65	23.249195548965	32.1302748861228	26.5900677144088	27.9422165736415	26.9111554708884	20.1380916938501	28.309642892044	25.4630870873728	29.9732678839124
RpbHLH10	5.46840166869081	3.80803257909603	13.382077201312	21.622542602969	46.2411282894533	27.8507143350109	35.6935626905593	28.8651296288241	31.6106584413647
RpbHLH106	42.1621026067166	27.8006633766453	32.1497654361424	16.2708706438092	20.3706694781233	20.86732772	20.1918391033679	21.7193326028954	18.1867661462477
RpbHLH8	27.6667254160295	25.035866364805	28.6431338818769	25.0621887784258	23.8349108557351	23.7240017745226	19.4172161198629	21.5166342037543	18.2521482857977
RpbHLH31	39.0801220243667	24.0762859813347	26.3987397484184	14.8488456074418	20.2167966099624	20.0345461184111	23.0610994399565	23.0201976861903	22.3532513263936
RpbHLH108	9.01408816992805	48.2959091675232	17.6935253005485	5.56340276368272	2.90832096658061	4.99785801653097	31.0402159299496	33.8944198626843	29.3155693265378
RpbHLH73	22.0516348546133	15.2482660476515	21.0912718414573	15.1630489938083	18.9715288568064	20.4045692777007	17.8543770512323	19.3519461864033	16.729265795672
RpbHLH104	21.1368891513234	14.5607359260741	13.6820251441894	15.3379136099001	15.4674607499802	15.5619805881876	14.1247351922868	13.7935746645067	14.965833969321
RpbHLH27	15.9684059966788	9.66079211103018	17.7544076761448	12.2686187634929	10.0784129441556	7.92938003489712	15.469609164413	12.8837397502897	13.4853108870617
RpbHLH20	20.9398000525373	13.6341715725712	17.1594752040382	8.9025429218098	6.17361494649942	7.43837151320708	10.7703400257747	13.7406374261473	15.5156004597876
RpbHLH64	24.5947415526588	24.8325468362778	21.3098834919096	7.85545450165421	4.9387034882381	6.94354839938707	6.17459433149128	6.78068230276215	4.80784011895475

Continued Table S6. Flower Color and *RpbHLH* Gene Expression (FPKM)

	White_1	White_2	White_3	Pink_1	Pink_2	Pink_3	Purple_1	Purple_2	Purple_3
RpbHLH12	18.1808358809421	27.9111478808743	15.529561663297	8.07875857971225	5.08968687750809	3.87099065934444	7.63886337291241	9.10498265548481	9.89665790719905
RpbHLH47	2.68358928209536	4.15986167607774	3.02723068407667	11.829353103417	17.3654350520355	18.8796333734295	14.5103237962392	14.939479902277	11.5700058625226
RpbHLH78	23.1033628625294	14.2901082710309	11.4679839975117	8.77141555232627	9.41358600463882	8.95268845643819	8.66060455528025	8.33994860987602	8.12033469308641
RpbHLH52	10.5578052019278	11.0346398598805	12.2553071987862	10.3408109820317	9.18945300452837	11.9788018645208	11.5751247344838	9.10498265548481	10.1165836384701
RpbHLH39	8.48306989130294	4.76967643383739	6.47742356727232	12.1353756190272	13.1757536907033	14.0035588598397	8.26066939836166	5.4272615392706	11.2634028363534
RpbHLH97	12.432399697739	10.2699508768429	10.1973822883171	6.94874932705943	6.501392161	5.92273807941847	8.28764354978991	9.18173698767013	10.4389405322511
RpbHLH26	8.74611081816504	5.55338084451505	5.57111738913546	7.75142795950401	8.85038000436128	9.91834556090127	11.6937210647782	8.62608810424299	10.8208472337656
RpbHLH57	14.7510936102289	10.8558071610709	14.2850323313952	7.68573789205057	3.18665285871317	4.27814352304315	7.42427825535401	9.43373399155411	6.00681504117977
RpbHLH15	9.58590131826989	6.53140014078561	7.79562055826231	7.25735250134612	5.09259570742755	6.70125596109464	11.0459290308097	12.806680521977	9.51809722222423
RpbHLH90	5.94900071523631	7.81586933672488	7.10223677259735	7.40108093308574	5.74936502340929	7.27108343628321	10.2517207793334	9.79671922950074	9.52408139400318
RpbHLH101	7.28177137264253	2.49633714654087	7.10074706531341	6.80447847209741	5.32947829209994	7.00827458709042	11.7289155618765	8.81930000131474	8.32162588437414
RpbHLH102	7.85511358024894	3.8626559972388	5.6463606847227	5.60680879010248	8.95919615195587	6.44351534720638	7.90847031548579	5.93603535042325	8.19613927469308
RpbHLH45	5.56028681237819	5.4217061356943	7.09970717069804	5.54903897600332	4.19435614314573	6.80331329961115	4.43005598585303	3.45251177616213	5.11943299763824
RpbHLH107	4.09662965629602	4.35986030903141	4.32624381324458	4.99913039881166	4.25456004634434	7.35424229514276	5.87886288938767	4.9088810504724	5.1380878810237
RpbHLH16	14.6565685195032	5.06219174638132	5.94589670390973	3.73779049828241	3.46705734545849	2.89526705221572	3.52174068736477	3.47592562013025	3.77997350622186
RpbHLH49	5.80924950886254	3.27782163725571	11.4664356001314	8.12335997843068	4.91337822306178	6.95388631370092	0.0844320674251152	1.06926842573309	0

Continued Table S6. Flower Color and *RpbHLH* Gene Expression (FPKM)

	White_1	White_2	White_3	Pink_1	Pink_2	Pink_3	Purple_1	Purple_2	Purple_3
RpbHLH68	4.38892594999309	10.9327032329995	9.54534877879219	2.34299630889302	2.10674667540306	2.0229789522935	1.91775839059124	2.08173968335565	1.39675695380022
RpbHLH103	6.10615570945625	4.83681557425504	3.80461572432528	2.11691539954743	2.32475419469398	3.95701736529611	3.6500632225319	4.98957915707136	4.68228976254579
RpbHLH36	1.23504988182778	0.937749958273937	2.09923414188962	2.86886990440598	3.35166629197421	3.57086979544327	6.61468830534872	5.75153459485073	6.85621000944205
RpbHLH67	4.1509319597323	3.53036353687027	2.79822032499758	4.22715584062782	4.24607516875904	4.07612007889944	3.11945217999717	3.46689724189614	2.55358210198099
RpbHLH23	11.6539579429906	2.56918383000789	4.07816112479459	1.13286888004214	1.99517237092537	1.59039367240297	1.81711962451191	2.93918070603911	1.88780179732698
RpbHLH89	5.90419969999266	5.84784144144157	4.56692581010522	0.778004142446838	0.302510797695083	0.688964831164924	3.56608937538777	2.19782851735746	1.92940978967889
RpbHLH80	16.1923338261769	5.32178466022737	5.31375598800693	0	0	0	0	0.143556821610606	0
RpbHLH50	1.74029756075733	8.4846180435216	2.30869209396287	1.64694383401084	0.640380000315565	0.364614504804814	1.23248888033544	1.27569811931243	1.64944298453318
RpbHLH29	0.527743664633993	0.541304270042982	0.160441598587672	3.66251769585082	2.22514711300986	2.35143448152823	0.342605212223211	1.00126996155845	0.96068640735747
RpbHLH13	1.97214158655806	6.93537130762853	1.46142545492632	0	0	0.0378756382731476	0.480109496386995	0.623612769762092	0.448752335308633
RpbHLH99	0.72728853285381	0.289256206034746	0.394832292188923	1.83540009362402	1.87494343375975	2.02732920681523	0.674496402173628	0.9691870991323	1.51306402182242
RpbHLH34	1.14654898120483	0.644005509700064	1.16189079238655	1.53714757841012	1.03130478482193	0.880794254744177	1.11648992689211	0.755315303245345	0.948699232934115
RpbHLH93	4.06069430843378	1.97626690767818	2.84377958449072	0	0	0	0	0.206362931065247	0.0719994953566068
RpbHLH85	4.15691929204595	1.62427929914049	2.00095766200691	0.109275894199771	0.116846587735305	0.106446699033064	0.449770823629524	0.219077234874954	0
RpbHLH109	1.94413082615635	0.749656436913737	0.404398177681432	0.258223578267681	0.586741078217271	0.534518398644331	1.06282755523094	0.711822112195193	1.18532067017317
RpbHLH98	0.244129918342713	0.429262189527359	0.846096326018956	2.1717215286155	1.03756959970969	0.810189504864884	0	0.185271729693848	0.921131820353866

Continued Table S6. Flower Color and *RpbHLH* Gene Expression (FPKM)

	White_1	White_2	White_3	Pink_1	Pink_2	Pink_3	Purple_1	Purple_2	Purple_3
RpbHLH33	1.96955831726429	0.192396855605525	0.399182540198669	1.86390649477874	0.295265030085621	0.537970143616205	0.142068329020703	0.276798182985719	0.434583780356046
RpbHLH41	0	0	0	0.805307095306769	1.8128404420698	1.7340636890276	0.0436128877692231	0	0.0444702765437867
RpbHLH43	0.256464903690554	0.225475613235949	1.07597202589691	0.291249014856653	0.467140358124933	0.330993209414391	0.249741167857446	0.389265655230443	0.458371524122904
RpbHLH11	1.39361106443289	0.168995528658108	0.876574414374461	0	0	0	0.233978411700763	0.683806162109692	0.429440777038224
RpbHLH37	0	2.48750515247402	0.0716811658206214	0	0	0	0.306134334792998	0.0745569299332501	0
RpbHLH48	1.1914017530857	0.785581293059236	0.27165234	0	0.12056053795672	0	0.174025019167169	0.169530427476584	0.0591487296817114
RpbHLH22	0	1.65144270011818	0.755821816475938	0	0	0	0	0	0
RpbHLH2	1.0664449698917	0.649096462345915	0.261865538435603	0.155267432162638	0.0415061111315644	0.0756237491447021	0.0798835385402605	0.0389101822883967	0
RpbHLH5	0	0	0	0.329388766802167	0.422650800208273	0.192516458536942	0.271147553673799	0	1.03679273313514
RpbHLH100	0.30619238950488	0.346107090335254	0.0797887303568856	0.0827907133793598	0.265579497437874	0.201618074470167	0.340759941780357	0.290464161499377	0.130297111884129
RpbHLH21	1.47925292664373	0.459003926944611	0	0	0	0	0	0.165090344852197	0
RpbHLH83	0.770408406343166	0.507988535748976	0.526984064926306	0	0	0	0	0.0913543410249312	0
RpbHLH38	0.0660275497306304	0.116098554240733	0.0602199496053999	0.0624856739191103	0	0.121735791306106	0.192889519889897	0.125271806391911	0.590044644873656
RpbHLH62	0.620743078996246	0	0	0.146861233606062	0.0523452866499984	0.333804192084577	0.0503724223916293	0.147214320250367	0
RpbHLH95	0.181596267209461	0.399133849517053	0.165623563262553	0	0.459403043704644	0	0	0	0
RpbHLH28	0	0	0	0	0	0	0	0.455421640971578	0.715029471127682

Continued Table S6. Flower Color and *RpbHLH* Gene Expression (FPKM)

	White_1	White_2	White_3	Pink_1	Pink_2	Pink_3	Purple_1	Purple_2	Purple_3
RpbHLH82	1.18656651869818	0.0695460495370622	0	0	0	0	0	0	0
RpbHLH42	0	0.532840379537691	0	0	0	0.111742554706351	0.118036870380385	0	0
RpbHLH24	0	0	0	0	0.503155714533659	0	0.0691702943045404	0.134767628450774	0.0705301179003496
RpbHLH7	0.270712953895585	0	0	0	0.136970166734163	0	0.26361567718286	0	0
RpbHLH84	0.461879921338439	0.10151745619628	0	0	0	0	0	0.109538617437477	0
RpbHLH96	0	0.149791491310596	0.0776963685468272	0.322478512953171	0	0	0	0	0
RpbHLH91	0.468541650973128	0	0	0	0	0	0.152085967605496	0	0
RpbHLH81	0.189604403506636	0	0	0.269150354196324	0	0	0	0	0
RpbHLH74	0	0	0	0.0866812544216231	0	0.25331112965387	0	0	0
RpbHLH44	0	0.256018126582652	0	0	0	0	0	0	0
RpbHLH56	0	0	0.207674405648529	0	0	0	0	0.0720020195617059	0
RpbHLH72	0	0.218573298545053	0	0	0	0	0	0	0
RpbHLH9	0.253134190655612	0	0	0	0	0	0	0	0
RpbHLH3	0	0	0	0	0	0.174787964949233	0	0	0
RpbHLH88	0.164068456906415	0	0	0	0	0	0	0	0
RpbHLH75	0.0963010507928959	0	0	0	0	0	0	0	0

Continued Table S6. Flower Color and *RpbHLH* Gene Expression (FPKM)

	White_1	White_2	White_3	Pink_1	Pink_2	Pink_3	Purple_1	Purple_2	Purple_3
RpbHLH53	0	0	0	0	0	0	0	0	0
RpbHLH54	0	0	0	0	0	0	0	0	0
RpbHLH69	0	0	0	0	0	0	0	0	0
RpbHLH18	0	0	0	0	0	0	0	0	0
RpbHLH25	0	0	0	0	0	0	0	0	0
RpbHLH105	0	0	0	0	0	0	0	0	0
RpbHLH55	0	0	0	0	0	0	0	0	0
RpbHLH59	0	0	0	0	0	0	0	0	0
RpbHLH60	0	0	0	0	0	0	0	0	0
RpbHLH1	0	0	0	0	0	0	0	0	0
RpbHLH92	0	0	0	0	0	0	0	0	0
RpbHLH94	0	0	0	0	0	0	0	0	0
RpbHLH77	0	0	0	0	0	0	0	0	0
