# Supplementary material for: Identification and Expression Analysis of the bHLH Gene Family in Rhododendron × pulchrum Sweet with Different Flower Colors
Source: Plants (Basel). 2025 Jun 4;14(11):1713. doi: 10.3390/plants14111713 (PMC12157016; doi:10.3390/plants14111713)
Supplement: Supplementary file 1 [file plants-14-01713-s001.zip › Table S7 ANOVA results for the RpbHLH genes.pdf]

Table S7. ANOVA results for the *RpbHLH* genes

Gene	Degrees of Freedom (df)	F-statistic	p-value
RpbHLH4	8	10.338	0.000
RpbHLH6	8	57.086	0.000
RpbHLH10	8	46.449	0.000
RpbHLH14	8	31.749	0.000
RpbHLH15	8	11.450	0.000
RpbHLH17	8	22.463	0.000
RpbHLH26	8	24.251	0.001
RpbHLH36	8	152.259	0.000
RpbHLH46	8	4.403	0.004
RpbHLH47	8	351.969	0.000
RpbHLH58	8	22.463	0.000
RpbHLH70	8	101.966	0.000
RpbHLH71	8	35.990	0.000
RpbHLH90	8	45.464	0.000

All genes demonstrate statistically significant differences, with $p<0.05$.
