# Supplementary material for: Identification and Expression Analysis of the bHLH Gene Family in Rhododendron × pulchrum Sweet with Different Flower Colors
Source: Plants (Basel). 2025 Jun 4;14(11):1713. doi: 10.3390/plants14111713 (PMC12157016; doi:10.3390/plants14111713)
Supplement: Supplementary file 1 [file plants-14-01713-s001.zip › Table S8 qRT-PCR Primer Sequences for bHLH.pdf]

Table S8. qRT-PCR Primer Sequences for *RpbHLH*

Primer name	Primer sequences (5'-3')
UBQ-F	CACCACACAAGACACAGATACT
UBQ-R	GGAAGGACTTTGGCTGACTATAA
bHLH4-F	GCCAAAGTGCTGATGTGCAA
bHLH4-R	GGCTTTCGTTCCCAATCTGC
bHLH6-F	AAGATATGGGGGCTGCGATG
bHLH6-R	AAGTTCTGGGGCATCTGGTG
bHLH10-F	AGCTCAACCACCGCTTCTAC
bHLH10-R	CTGCTGTGGTTATCGGTGGT
bHLH14-F	GGCTCAACAAAAACGGTCCC
bHLH14-R	TTGGCACCAGTTTTCCCTGT
bHLH15-F	AACGGCTTCACAGATGCTGA
bHLH15-R	GGCAGTGACAGAGGCTCTTT
bHLH17-F	GGTCCTCCACATCCCACAAG
bHLH17-R	CCGGCCCGAACAACTATTCT
bHLH26-F	AAGTCTGAAGAGCCACCACG
bHLH26-R	GCCCTTCTTACCAGTGCCTT
bHLH36-F	GGAGGGGGAAGAAATGTGGG
bHLH36-R	GCCCCAAATGATGATGACGC
bHLH46-F	TCAATGAGACTTGCGGCTGT
bHLH46-R	GGGTGTGATTGGACGATGGT
bHLH47-F	TGGGTTGTTGAGGTTTCGCT
bHLH47-R	TGCCGCATGAGATGATTCGA
bHLH58-F	CCTTCCCCCTCTGTAGTCGA
bHLH58-R	TCTGGTGGGGATTCGAGCTA
bHLH70-F	GGTCGGATTCAGTGTCGGTT
bHLH70-R	GCTCTTCTTCCCAACCCCAA
bHLH71-F	CTCATCCACGAAGCATTGCG
bHLH71-R	ATCCAACATGTCCGCTGTGT
bHLH90-F	AGGTTAGCGTGGACAAGAGC
bHLH90-R	CAAACGTCGCTTCCCTCTCT
